# Deliberation as a condition for meaningful participation in health and healthcare research

**DOI:** 10.3389/fpubh.2026.1709598

**Published:** 2026-03-03

**Authors:** Jesse David Marinus

**Affiliations:** 1 Campus Fryslân, University of Groningen, Groningen, Netherlands; 2 Planbureau Fryslân, Leeuwarden, Netherlands

**Keywords:** citizen science, community based participatory research, deliberation, epistemic justice, participation, participatory health research, patient participation

## Introduction

*The participatory approaches in health and healthcare research have moved “non-academic participants”*^1^ *from passive subjects to active partners. However, a persistent critique remains, that despite the increase in the quantity of participation, the “meaningfulness” stays behind. Researchers struggle to define what makes participation meaningful beyond the presence of non-academic voices. In this opinion paper I argue that meaningful participation is not a performative checklist, but a standard of epistemic justice and legitimacy, where the input of non-academic participants is not downgraded but are part of the core work of science to produce collective knowledge. To bridge the gap between tokenistic practices and meaningful participation, we need a mechanism that allows for epistemically just and legitimate knowledge. I propose that this mechanism is genuine deliberation, where problems and solutions are understood collectively by listening to another, critiquing each other's claims, and engaging in an open and reflective process*.

In recent literature reviews, the participation of non-academic participants in participatory health and healthcare research^2^ often remains limited. In many studies, their role in deciding the study design and analyzing the findings is minimal (1, 2). For example, communities are frequently invited to assist with recruitment or implementation of interventions, but rarely with setting the research agenda or interpreting findings (3–6). This reveals a pattern whereby participation remains technical and researcher-controlled, or in other words, where researchers define the boundaries, and participants merely fill in the blanks.

Recent critiques reinforce these concerns, warning that this technical focus risks hollowing out the participatory process. Turcotte et al. (7) argue that the “patient-oriented” research paradigm is often depoliticised by existing power structures, in ways that betray more critical forms of participatory research. They explain this as a conflict between the dominance of evidence-based science and participatory approaches that seek to generate alternative forms of knowledge capable of challenging the dominant system.

From their perspective, participation is not an inevitable “evolution” of research, but a disruption of it. Others offer a milder, yet equally critical perspective (8), warning that patient participation may become tokenistic and instrumental, used to satisfy funding and governance requirements while inadvertently reproducing power imbalances.

This technical reductionism stands in sharp contrast to the historical and philosophical roots of participatory research. Foundational thinkers such as Kurt Lewin and Paulo Freire emphasized that participation must challenge power inequalities and promote the democratization of knowledge (9–11). Similarly, Arnstein made the threshold of meaningfulness explicit: participation without a redistribution of power is an empty ritual that preserves the status quo. In her analysis, citizen participation is best understood as citizen power, a deliberate shift in decision-making authority (12). The legacies of these scholars frame participatory research not as a method, but as a collective practice from beginning to end.

The disconnect between these ideals and current practice is particularly critical in the context of health and healthcare. While terms like co-creation and empowerment are frequently employed, the reality often remains tokenistic (3, 13). This is not merely a methodological flaw but a source of harm, as the exclusion from the creation of knowledge can lead directly to exclusion from care and treatment. Historically, marginalized communities have been subject to such epistemic injustice, where their symptoms are dismissed, their experiences pathologised, and their knowledge ignored (14). Recognizing this danger, international frameworks—Alma-Ata Declaration (15), the Ljubljana Charter (16), the Declaration of Astana (17), and the resolution on Social participation for universal health coverage, health and well-being (18)—have classified participation not just as a preference, but as a necessity.

At times, I would argue that participation in some studies is performative, where participatory language seems to be used to secure funding or legitimacy for otherwise traditional (positivist) research. In such cases, I consider participatory language to function as a cloak of consent, where outputs appear endorsed by non-academic participants while their influence is superficial. This is epistemically harmful because it reproduces epistemic injustice, meaning that voices are present but structurally unable to shape framings, interpretations, and recommendations (19). It is also a problem of legitimacy, because findings and recommendations may be implemented under the illusion that marginalized voices have meaningfully contributed to and endorsed them while they were not part of agenda setting and interpretation of findings.

Yet expanding participation to collective agenda setting and collective interpretation is not straightforward. It is hard precisely because these stages touch the core of scientific authority: who defines the problem, what counts as a valid interpretation, and whose reasons carry weight. Enabling participation here requires a redistribution of decision-making power, what Arnstein identified as true citizen power. In practice, this means researchers must be willing to let non-academic participants challenge claims, reorient priorities, and shape interpretive outputs, which confronts entrenched hierarchies of expertise and the institutional expectation that researchers retain control over core decisions.

Consequently, the participation of non-academic actors has become a largely technical issue rather than a matter of “meaningfulness”. This leads to the question: when is participation ‘meaningful'? In other words, when does it move beyond superficial gestures hidden beneath a cloak of consent and tokenistic practices to become legitimate and epistemologically meaningful knowledge-making? In this opinion paper, I operationalise “meaningful participation” through epistemic justice^3^ and legitimacy^4^. I argue that deliberation, understood not as a technique but as a process for participants to question claims, demand reasons, and revise interpretation, offers a path toward greater epistemic justice and legitimacy in participatory health and healthcare research. Drawing on Jürgen Habermas's Between Facts and Norms (20) as a companion thinker, I reflect on how deliberation can strengthen participatory approaches and I provide three conditions that can plausibly advance epistemic justice and legitimacy rather than merely staging participation. This paper is not a conclusion. Instead, it offers a starting point from which to advance the discussion on participation in health and healthcare research.

## Habermas, deliberation, and systemic inequality

To address the problem of “meaningfulness”, Habermas's theory of communicative action can help explain why deliberation can function as a condition for meaningful participation. Deliberation is a process of collective sense-making in which participants can question claims, request reasons, and revise interpretations together. In Habermas's terms, rationality is not found in top-down technocratic systems, but in our ability to understand problems and possible solutions through dialogue [(20), p. 3–4, (21)]. For research to be accepted by society, the claims it makes should not be imposed as unquestionable truths but must be open to critique and intersubjective recognition [(20), p. 4]. A deliberative orientation therefore asks researchers not only to “collect” perspectives within a pre-set frame, but to treat framings, interpretations, and recommendations themselves as legitimate objects of discussion. This enables non-academic participants to shape the agenda and the interpretation, rather than merely contributing within a pre-defined frame.

When non-academic participants and academic researchers engage in an open and reflective process aiming to listen carefully, weigh reasons, and attend to voices that are often ignored, they enact a process of collective knowledge. This allows for new insights into the subject of study by giving space to novel perspectives. Furthermore, it strengthens perceived legitimacy because conclusions are seen as emerging from a fair process of mutual critique and justification rather than from expert authority alone. This process does not eliminate disagreement. Rather, differences are acknowledged and discussed [(20), p. 18]. It surfaces hidden assumptions and challenges framings or conclusions that may dominate within traditional (positivist) academic paradigms.

However, operationalising deliberation in research is complex. In practice, deliberation requires more than simply inviting people into a study. It requires time and careful design to create moments where claims (such as frameworks, interpretations, or recommendations) are jointly tested. This is what Habermas calls a “first-person plural” perspective [(20), p. 92], where knowledge is treated as a co-construction, rather than something one party delivers and the other receives. However, in practical terms, this does mean that “*one has the possibility of taking a yes or no position to a criticizable validity claim [but] only if the other is willing to justify the claim […]*” [(20), p. 119]. Deliberation therefore requires a protected space for participants to question, respond, and refine interpretations, especially when views diverge. Under these conditions, legitimacy is not derived from authority but is earned through conclusions that are collectively justifiable because they have been tested through reasoned contestation and mutual critique [(20), pp. 305–306]. Thus, meaningful participation entails an open and reflective exchange through which knowledge claims become legitimate because they are jointly tested and contestable.

Nevertheless, deliberation remains fragile. Habermas (and his critics) warn that the public sphere is shaped by societal structures that can produce exclusion, power imbalances, and distorted communication [(20), p. 307; see (21)]. But as even formally inclusive practices may privilege dominant groups, Fraser (22) argues that “subaltern counterpublics” can be needed. These are parallel spaces in which subordinated groups can develop and articulate their interpretations of needs and interests. Because for fair deliberation, non-academic participations should also be able to develop their own perspectives and claims. Likewise, Foucault (23) emphasizes that power is not merely held by particular actors but is diffused and woven into discourse itself, meaning that deliberative spaces must continually guard against subtle forms of domination. If research with deliberation ignores these realities, it risks slipping back into paternalism [(20), p. 303]. These distortions are not only political but epistemic. They can take the form of testimonial injustice, where some participants' contributions are treated as less credible, and hermeneutical injustice, where people lack the shared concepts or safe platforms to articulate lived experience in ways that can shape interpretation (19). Deliberation must therefore accommodate multiple modes of communication and recognize different standpoints as legitimate contributions to collective reasoning (24). Aiming to ensure that those intended to participate are not structurally excluded.

To avoid epistemic injustice, deliberation must thus be designed intentionally, with measures that push back against power differences and allow for needs and interests to be interpreted in diverse ways, rather than through a single dominant framing. This keeps pluralism alive while still aiming for reasoned agreement and contestation [(20), p. 306]. Yet even with such safeguards, securing epistemically just and legitimate collective knowledge requires confronting how power distorts whose claims are heard and taken up. Tacit hierarchies and imbalances of skills, such as differences in the ability to articulate arguments or debate, can create gaps between academic and non-academic participants. Thus, the fragility of deliberation requires ongoing rethinking of positionality and systemic inequality to continuously adjust the conditions under which dialogue unfolds.

## How to move forward

These ideas on deliberation stand in sharp contrast to the view that participation is simply a box to be ticked or a ladder to be climbed. Too often, I see that participatory research relies on methods such as focus groups, workshops, or citizen panels as proof of participation. Yet, these methods, while potentially valuable, are not in themselves epistemically different from a survey or a biomedical test. These methods can facilitate inclusion, but they can equally reproduce tokenism or silence dissenting voices if they only offer procedural inclusion.^5^

What is required is an orientation from performative methods toward an orientation of reflexivity and inquiry. This orientation must reorganize who sets the questions, who holds decision-making authority, and how knowledge is owned and used (6, 25, 26). Furthermore, it demands a quality of public reasoning that provides genuine room for the contestation and justification of claims (27, 28). Based on these considerations, I propose that the foundation of meaningful participation lies in deliberation. Drawing on Habermas (20, 21, 29), I highlight three conditions for deliberation in participatory research that can plausibly advance epistemic justice and legitimacy.

First, non-coercion is essential to protect testimonial justice. Non-academic participants must be free to speak, question, and disagree. This requires more than the mere absence of intimidation. It demands the active creation of conditions where critique is possible, allowing participants to challenge dominant framings and conclusions. In practice, this can be achieved through, for example, protected speaking formats, the explicit legitimization of dissent, and allowing space for off-topic discussions that may reveal hidden priorities

Second, equity is necessary to safeguard hermeneutical justice, ensuring that every voice carries the potential to shape agendas and interpretations. This does not imply that all perspectives are identical or that differences in expertise should be erased, but rather that doctors, researchers, and community members possess equal standing. Each party brings different forms of knowledge to the table to introduce concerns and question the assumptions guiding collective decisions. To achieve this, researchers must address uneven resources (such as time, money, and language barriers) and allow for non-academic participations to develop their own voice, for example, by creating parallel spaces for subordinated groups to articulate their views.

Third, rational justification is the basis for legitimacy. Decisions and knowledge claims must remain open to reasoned challenge and mutual understanding between academic and non-academic participants. Only under such conditions can genuine collective sense-making occur, making the outcomes publicly defensible rather than authoritatively imposed. In practice, this could entail explicit “reason-giving” (explaining the “why”) throughout the process and accurately reporting disagreements as part of the research output.

Crucially, these conditions and design moves should not be mistaken for another simple checklist, which would risk falling into the same trap as previous approaches. Instead, they should be viewed as support for developing an active stance that helps academic researchers rethink their positionality, their relationships with one another, and the nature of the knowledge they produce.

## Conclusion

In this paper, I have argued that “meaningful” participation in health and healthcare research is conditional upon genuine deliberation. Drawing on Habermas, I suggest that legitimacy and epistemic justice do not arise from the mere presence of non-academic voices, but only when research is open to critique, when participants can contest validity claims, and when knowledge-making is treated as a truly collective act. I take the position that deliberation, despite its fragility, makes participation epistemologically just because it allows everyone involved to contribute and to critique, and it grounds legitimacy not in the authority of experts, but in the quality of justification.

Moving forward, the field must resist the temptation to reduce participation to a performative checklist. Instead, by prioritizing the conditions of non-coercion, equity, and rational justification, researchers can move beyond tokenism toward a form of inquiry that is epistemically just. Ultimately, this paper serves not as a final conclusion, but as a departure point. It is an invitation to rethink the nature of participation, ensuring that the knowledge we produce is not only scientifically robust but also socially just and legitimate in the eyes of those it affects.

## Footnotes

1 I use this term to categorise the big of group of non-formally trained researchers such as citizens, patients, healthcare professionals, and more who are involved in research projects.

2 Such as citizen science, patient involvement, participatory action research, or community-based participatory research.

3 Epistemic justice concerns whether those affected can genuinely shape what counts as knowledge, rather than having their contributions discounted or confined to data provision (19).

4 Legitimacy concerns whether research claims are publicly defensible because they emerge through reason-giving and contestation rather than expert authority alone (27, 28).

5 To be clear, I am not arguing that all academic research must be participatory. However, when a participatory approach is adopted, it should be pursued with genuine meaningful participation. Treating participation as a checkbox and using a single focus group to gather non-binding advice risks epistemic harm. If participation is limited in scope, this should be documented transparently and honestly, rather than wrapped in the cloak of consent.
